# The relationship between the different low birth weight strata of newborns with infant mortality and the influence of the main health determinants in the extreme south of Brazil

**DOI:** 10.1186/s12963-019-0195-7

**Published:** 2019-11-27

**Authors:** Cássia Simeão Vilanova, Vânia Naomi Hirakata, Viviane Costa de Souza Buriol, Marina Nunes, Marcelo Zubaran Goldani, Clécio Homrich da Silva

**Affiliations:** 1 Postgraduate Program in Child and Adolescent Health, Faculdade de Medicina, Universidade Federal do Rio Grande do Sul (UFRGS), Porto Alegre, Brazil; 2 Research Group and Graduate Studies, Hospital de Clinicas de Porto Alegre (HCPA), Porto Alegre, Brazil; 3 Department of Pediatrics, Faculdade de Medicina, Universidade Federal do Rio Grande do Sul (UFRGS), Rua Ramiro Barcelos 2400/Sala 414, Porto Alegre, RS 90035-003 Brazil; 4 Pediatrics Service, Hospital de Clinicas de Porto Alegre (HCPA), Porto Alegre, Brazil

**Keywords:** Infant mortality, Weight at birth, Risk factors, Information Systems, Maternal and child health

## Abstract

**Background:**

Low birth weight (LBW) newborns present different health outcomes when classified in different birth weight strata. This study evaluated the relationship of birth weight with Infant mortality (IM) through the influence of biological, social, and health care factors in a time series.

**Methods:**

Retrospective cohort study with data collected from Information Systems (Live Births and Mortality). The mortality trends were performed for each birth weight stratum: extremely low, < 1000 g; very low, 1000–1499 g; low, 1500–2499 g; insufficient, 2500–2999 g; adequate, 3000–3900 g; and macrosomia, > 4000 g. Chi-square tests analyzed IM rates. Sequential Poisson regression analyzed the impact of the determinant factors.

**Results:**

A total of 277,982 newborns were included in the study and 2088 died before their first year. There was a tendency for a decrease in mortality in all strata of weight. With the exception of macrosomics, all other strata had a higher risk for IM when compared with adequate birth weight. Extremely LBW newborns presented higher risk for mortality when born in a public hospital. A higher percentage of infant deaths were associated with lower maternal age and lower schooling for all strata. Prenatal care with less than three visits demonstrated a risk for IM in low, insufficient, and adequate birth weight strata. The cesarean section was a protective factor for IM in Extremely and Very LBW strata and it was a risk factor in adequate birth weight stratum.

**Conclusions:**

LBW had a greater association with IM, especially those children of younger mothers and those born in public hospitals.

## Background

Birth weight is an important indicator and prognostic factor for the health of newborns, as it reflects the nutritional and metabolic conditions of the mother, as well as fetal development during pregnancy. The World Health Organization (WHO) [1] defines birth weight as the first measurement obtained from the newborn and that defines the classification of weight strata. According to this measure, newborns are classified as “macrosomic,” a term used for newborns weighing more than 4000 g; “adequate birth weight” (ABW), which refers to the birth weight of 3000–3999 g; “inadequate or insufficient birth weight” (IBW), indicating the range between 2500 and 2999 g; and “low birth weight” (LBW), if the weight is less than 2500 g. It should be noted that the last classification includes two complementary and non-exclusive categories: “very low birth weight” (VLBW), when the weight is less than 1500 g, and “extremely low birth weight” (ELBW), which indicate less than 1000 g [1].

Birth weight, besides the gestational age and sex of the newborn, has a close relationship with infant mortality and its components (neonatal mortality—NM and postnatal mortality—PNM). The lower the birth weight and the gestational age, the greater the chance of death in the first year of life [2].

Newborns weighing less than 2500 g have a higher risk of neonatal morbidity and mortality, malnutrition in the first year of life, susceptibility to infections, respiratory distress and traumas during childbirth, and development of chronic non-communicable diseases (NCDs) [2, 3]. The estimated relative risk of low birth weight for neonatal mortality is almost 200 times higher when compared with newborns with adequate birth weight [4, 5].

In this context, a better understanding of the risk factors and outcomes associated with low birth weight allows a more qualified care to pregnant women and the newborn using more appropriately the new technologies developed in the area of prenatal, perinatal, and neonatal care. Lin et al. [6] highlighted the latest advances in perinatal and neonatal care, such as surfactant replacement therapy, mechanical ventilation, and neonatal intensive care centers, which have contributed to significantly lower infant mortality rates for newborns, in particular those with very low birth weight. These new technologies allow the survival of these fetuses, which would probably become stillbirths.

Despite these technological advances, maternal and child health conditions are related to the social reality and public care provided to this type of population. In this sense, health determinants that involve the various biological, social, and care-related aspects should be considered. Differences in exposure and vulnerability to health conditions such as living conditions, work conditions, food availability, types of behaviors, lifestyle, and the health system itself—especially when there is no access to protection factors such as prenatal care—are considered important determinants of health [7].

To investigate these several factors in Brazil, several researchers have used the Health Information Systems (SIS), such as the Live Birth Information System (SINASC) and the Mortality Information System (SIM). These provide information on vital statistics (birth and death) that are relevant for assessing the health conditions of a given population.

Low birth weight alone can be considered an important predictor of child mortality risk, and its temporal evaluation at a given location is relevant for the monitoring, planning, and execution of public policies in the field of maternal and child health. Thus, the purpose of this study was to analyze the association of the various birth weight strata of newborns, especially LBW and its categories, with infant mortality, observing the influence of the main determining factors in the municipality of Porto Alegre, the state capital located in the extreme south of Brazil.

## Methods

This study is characterized as a retrospective cohort study with secondary data on live births and deaths in the first year of life, from 2000 to 2015 in Porto Alegre, capital of the state of Rio Grande do Sul. The population of the municipality, according to the latest Brazilian Institute of Geography and Statistics (IBGE) Census, held in 2010, totaled 1,409,351 inhabitants, with an estimated 1,484,941 inhabitants for 2017 [8].

Information on all single live births of mothers residing in the municipality of Porto Alegre was collected from the Live Birth Information System from 2000 to 2014. Those newborns weighing less than 500 g, multiple births or with congenital anomalies were excluded from the analysis.

Birth weight was categorized into strata, based on the WHO definitions with the following denominations: extremely low birth weight (ELBW), 500–999 g; very low birth weight (VLBW), 1000–1499 g; low birth weight (LBW), 1500–2499 g; insufficient birth weight (IBW), 2500–2999 g; adequate weight (ABW), 3000–3999 g; and macrosomic (MACR), 4000 g or more [1].

For this survey, the biological, social, and health care determinants were categorized in order to analyze the relationship between birth weight and infant death. As for biological determinants, five variables were used: maternal age (10–17 years, 18–34 years; and 35 years or more), gender of the newborn (female or male); gestational age (less than 22 weeks; 22–27 weeks, 28–31 weeks, 32–36 weeks; and 37 weeks or more); the 5-min Apgar Index (less than 7 or greater or equal to 7); and skin color of the mother (white or black/brown/Asian/indigenous). Regarding the social determinants, three variables were used: maternal schooling (up to 8 years of study; 8–11 years; and 12 years or more); number of previous living children (none, 1; 2; 3 children or more); and number of previous deceased children (none; equal to; or greater than 1). For the care determinants, three variables were considered: number of prenatal visits (none; 1–3; 4–6; and 7 visits or more); type of delivery (vaginal or cesarean); and type of hospital (public, private, or mixed). In addition to the information obtained from the Live Birth Information System, the data on the occurrence and death period, from 2000 to 2015^1^, were investigated through the Mortality Information System. The databases, in the annual format, of these systems were provided by the Coordinator-General of Health Surveillance of the Municipal Health Department of Porto Alegre.

Through the number of the Live Birth Statement (LBS), present in both information systems, a linkage was developed. The non-convergent data by the LBS number was manually linked by the mother’s name and date of birth. Data that did not present convergence of information was considered as missing values and excluded from the survey.

Based on the number of live births and annual deaths presented in these two databases, a temporal analysis of the mortality trends for each weight stratum in the period studied (2000–2015) was performed. Preliminarily, the Average Annual Percentage Change (AAPC) was calculated, with a 95% confidence interval. This temporal trend was performed through the Join Point program (version 4.1.1.1). Subsequently, chi-square tests for trend were carried out to evaluate infant mortality rates.

In order to evaluate the impact of the determinants in each weight stratum on the outcome studied (infant mortality rate), univariate robust Poisson regression was performed considering all the available data. The reference categories^1^ were maternal age of 18–34 years; gender: female; gestational age according to the weight strata (500–999 g, 28–31 weeks; 1000–1499 g, 32–36 weeks; equal to or greater than 1500 g, ≥ 37 weeks); 5-min Apgar Index equal to or greater than 7; skin color of the mother: white; maternal schooling equal to or greater than 12 years; number of previous living children: none; number of previous deceased children: none; number of prenatal consultations equal to or greater than 7; vaginal delivery; and private hospital.

Afterwards, multivariable analysis was conducted using Poisson regression with robust variance. All variables that presented *P* < 0.20 were included in the model, with a 95% confidence interval, to evaluate the impact of the determinants and the incidence ratio (IR), for each of the weight strata, on infant mortality.

The database processing and analyses were performed by the Statistical Package for the Social Sciences (SPSS) - version 18.

## Results

From January 1, 2000, to December 31, 2014, there were 291,039 live births, born to mothers living in the municipality of Porto Alegre, according to Live Birth Information System data, and from 2000 to 2015, there were 3393 deaths of children under 1 year of age, according to the Mortality Information System. From this list, 129 (0.04%) birth records did not allow linkage with the death records and, therefore, were considered losses. A total of 13,057 newborns were excluded thus distributed among the exclusion criteria of the study: 280 who had a birth weight of less than 500 g, 7045 multiple births, and 5732 had congenital anomalies. The losses and exclusions included 13,186 newborns, corresponding to 4.53% of all births occurred during the study period. Thus, the final sample totaled 277,982 newborns, of which 2088 (0.75%) died before completing their first year of life.

**Fig. 1:**
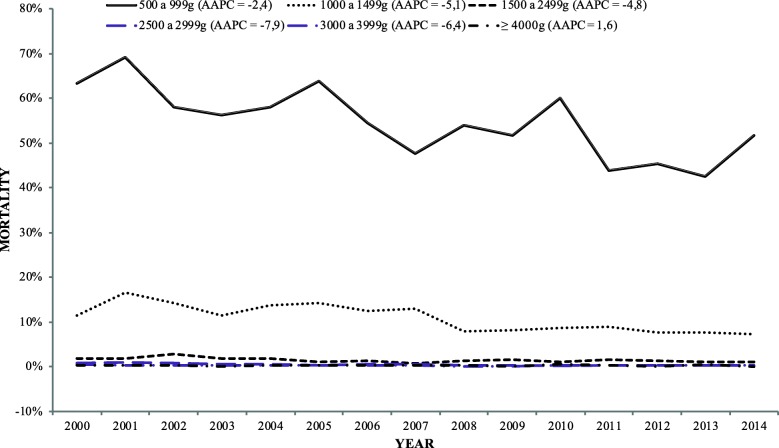
Percentage of mortality per year among newborns distributed by weight strata (500–999 g and 1000–1499 g) in Porto Alegre (2000–2015)

**Fig. 2:**
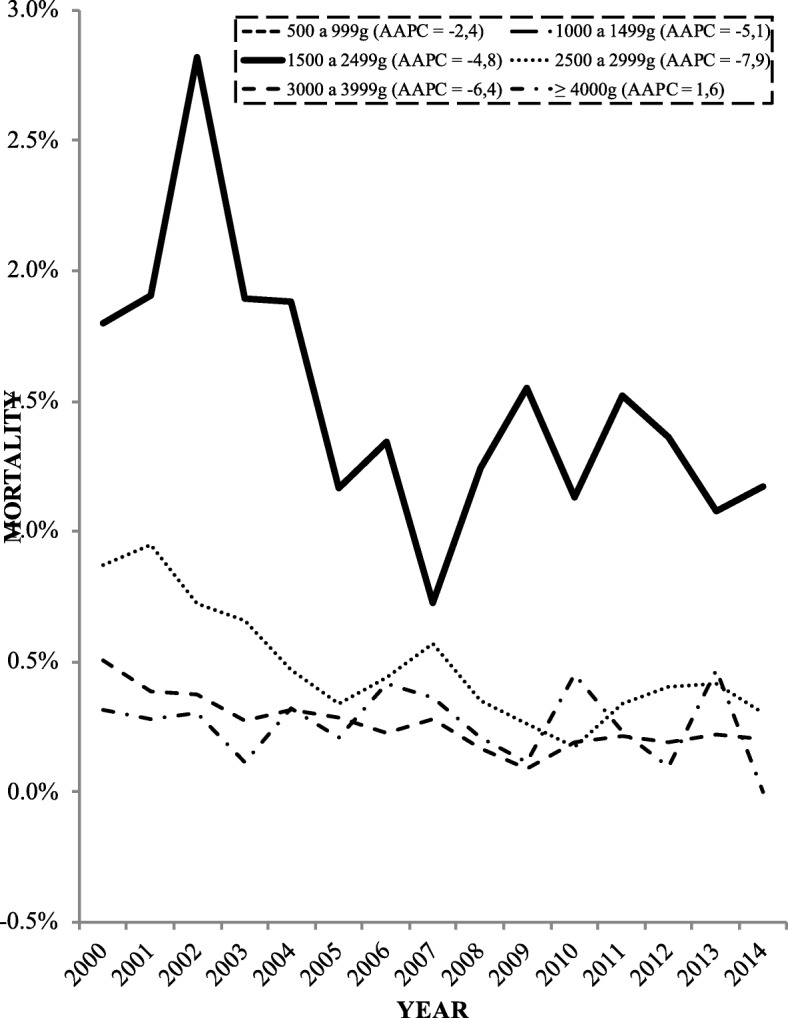
Percentage of mortality per year among newborns distributed by weight strata (1500–2999 g, 2500–2999 g, 3000–3999 g, and > 4000 g) in Porto Alegre (2000–2015), AAPC, Average Annual Percent Change

During the study period, with the exception of macrosomic newborns, there was a tendency for infant mortality rates to decrease in all strata. The lowest annual variation pattern (Average Annul Percent Change = 2.4) occurred among the newborns with extremely low birth weight (500–999 g), while the highest (AAPC = 7.9) occurred among those with insufficient birth weight (2500–2999 g) (Figs. 1 and 2).

Regarding the risk for infant mortality, with the exception of macrosomic, all other newborns with strata weighing less than 3000 g presented higher risk when compared with those of adequate birth weight (3000–3999 g). Newborns with extremely low birth weight were 200 times more likely to die in the first year of life. As the birth weight stratum increased, as predicted, the incidence ratio for the risk of death gradually decreased (Table 1).

**Table 1:** Percentage and incidence rate of mortality among newborns according to birth weight strata in the municipality of Porto Alegre (2000–2015)

Total	Mortality						*P*	IR	CI 95%	
	No		Yes		Total					
	*N*	%	*N*	%	*N*	%				
500–999 g	596	45.0	729	55.0	1325	100	< 0.001	207.04	186.81	229.47
1000–1499 g	1884	89.1	231	10.9	2115	100	< 0.001	41.1	35.31	47.83
1500—2499 g	19,536	98.5	304	1.50	19,840	100	< 0.001	5.76	4.99	6.65
2500—2999 g	64,032	99.5	319	0.50	64,351	100	< 0.001	1.86	1.61	2.15
3000—3999 g	17,5275	99.7	467	0.30	175,742	100	< 0.001	1	─	─
≥ 4000 g	14,571	99.7	38	0.30	14,609	100	0.899	0.97	0.7	1.36
Total	275,894	99.2	2088	0.80	277,982	100	─	─	─	─

Subsequently, the relationship between infant deaths and health care determinants was analyzed considering all the available data (Table 2). Among the biological aspects, the lowest maternal age (10–17 years) was associated with a higher percentage of infant death in the first year of life for newborns of all birth weight strata, even among those with adequate birth weight. Conversely, maternal age equal to or greater than 35 years, for newborns of this weight stratum, was shown to be a protective factor for infant death.

**Table 2:** Incidence of infant mortality and adjusted incidence rates of the determinants analyzed according to birth weight strata

	Crude analysis		Adjusted analysis	Crude analysis		Adjusted analysis	Crude analysis		Adjusted analysis
	Mortality rate (%)	*P*	IR (CI 95%) adj*	Mortality rate (%)	*P*	IR (CI 95%) adj*	Mortality rate (%)	*P*	IR (CI 95%) adj*
	500–900 g			1000–1499 g			1500–2499 g		
Biological determinants									
Maternal age									
10–17 years	66.67	0.0153	0.93 (0.81;1.07)	19.67	0.0003	1.74 (1.15;2.65)	2.19	0.0106	1.40 (0.94;2.08)
≥ 35 years	42.37	0.0002	0.87 (0.74;1.02)	7.44	0.0512	0.78 (0.53;1.16)	1.48	0.8815	
18–34 years	56.79		1	10.79		1	1.44		1
Gender									
Male	59.85	0.0006	1.16 (1.05;1.27)	12.26	0.0563	1.29 (1.00;1.66)	1.72	0.0466	1.07 (0.85;1.35)
Female	50.37		1	9.66		1	1.37		1
Gestational age									
< 22 weeks	92.50	< 0.0001	1.59 (1.29;1.96)	+					
22–27 weeks	63.19	< 0.0001	1.43 (1.23;1.66)	23.18	< 0.0001	1.90 (1.27;2.84)	+		
28–31 weeks	35.60		1	11.24	0.0099	1.27 (0.93;1.73)	5.03	< 0.0001	3.06 (2.03;4.61)
32–36 weeks	+			7.48		1	1.62	0.0008	1.48 (1.13;1.94)
≥ 37 weeks	+			+			1.05		1
5-min Apgar Index									
< 7	75.68	< 0.0001	1.43 (1.29;1.59)	28.46	< 0.0001	2.90 (2.21;3.81)	11.36	< 0.0001	6.3 (4.61;8.71)
≥ 7	40.18		1	7.78		1	1.21		1
Skin color of the mother									
Black/Brown/Asian/Indigenous	62.86	0.0006	1.02 (0.92;1.14)	11.44	0.6284		1.82	0.0722	0.89 (0.68;1.18)
White	52.49		1	10.67			1.45		1
Social determinants									
Maternal schooling									
None/< 8 years	65.60	< 0.0001	1.11 (0.94;1.32)	15.07	0.0002	0.83 (0.55;1.25)	2.32	< 0.0001	1.65 (1.06;2.58)
8–11 years	54.70	0.0001	1.01 (0.86;1.19)	9.08	0.3674		1.18	0.0598	1.12 (0.72;1.72)
≥ 12 years	39.73		1	7.64		1	0.83		1
Previous living children									
1	51.05	0.7130		11.49	0.0320	1.41 (1.00;1.98)	1.56	0.0620	1.26 (0.91;1.75)
2	63.97	0.0060	1.13 (0.97;1.31)	15.94	< 0.0001	2.28 (1.55;3.35)	1.67	0.0560	1.20(0.79;1.80)
≥ 3	65.34	0.0001	1.07 (0.91;1.25)	15.00	< 0.0001	1.72 (1.13;2.63)	2.63	< 0.0001	1.43 (0.97;2.11)
Nenhum	52.34		1	8.12		1	1.18		1
Previous deceased children									
≥ 1	55.17	0.9570		7.80	0.0808	0.64 (0.40;1.03)	1.93	0.1333	1.19 (0.82;1.71)
None	54.96		1	11.35		1	1.49		1
Health care determinants									
Prenatal visits									
None	76.79	< 0.0001	1.03 (0.84;1.26)	16.86	< 0.0001	1.41 (0.84;2.37)	3.40	< 0.0001	2.38 (1.54;3.68)
1–3	61.48	< 0.0001	1.00 (0.82;1.21)	14.79	< 0.0001	1.65 (1.05;2.60)	2.70	< 0.0001	2.14 (1.47;3.12)
4–6	48.0	0.0012	0.92 (0.75;1.13)	10.95	0.0001	1.60 (1.02;2.49)	1.59	< 0.0001	1.49 (1.06;2.08)
≥ 7	34.55		1	4.82		1	0.85		1
Type of delivery									
Cesarean	37.93	< 0.0001	0.74 (0.66;0.84)	8.44	< 0.0001	0.75 (0.56;0.99)	1.46	0.4301	
Vaginal	72.34		1	16.02		1	1.60		
Type of hospital									
Public	65.86	< 0.0001	1.85 (1.41;2.43)	12.05	0.0004	1.56 (0.84;2.88)	1.79	< 0.0001	1.01 (0.62;1.64)
Mixed	47.66	< 0.0001	1.46 (1.10;1.94)	11.30	0.0015	1.45 (0.77;2.74)	1.57	0.0015	0.81 (0.49;1.33)
Private	26.73		1	4.72		1	0.83		1
	2500–2999 g			3000–3999 g			≥ 4000 g		
Biological determinants									
Maternal age									
10–17 years	0.58	0.4529		0.50	< 0.0001	1.56 (1.13;2.14)	0.30	0.9178	
≥ 35 years	0.40	0.1821	0.72 (0.51;1.03)	0.16	0.0023	0.63 (0.45;0.88)	0.155	0.2458	
18–34 years	0.50		1	0.26		1	0.28		
Gender									
Male	0.55	0.0886	1.11 (0.89;1.39)	0.30	0.0023	1.29 (1.07;1.57)	0.24	0.5527	
Female	0.45		1	0.23		1	0.29		
Gestational age									
< 22 weeks	+			+			+		
22–27 weeks	+			+			+		
28–31 weeks	+			+			+		
32–36 weeks	0.77	0.0002	1.48 (1.11;1.99)	0.56	0.0005	1.55 (0.92;2.29)	+		
≥ 37 weeks	0.45		1	0.26		1	0.26		
5-min Apgar Index									
< 7	6.81	< 0.0001	12.19 (8.44;17.61)	6.06	< 0.0001	22.28 (17.9;29.06)	9.32	< 0.0001	58.54 (28.67;119.50)
≥ 7	0.44		1	0.22		1	0.17		1
Skin color of the mother									
Black/Brown/Asian/Indigenous	0.82	< 0.0001	1.30 (1.02;1.67)	0.43	< 0.0001	1.28 (1.05;1.57)	0.37	0.1162	1.53 (0.76;3.09)
White	0.40		1	0.22		1	0.21		1
Social determinants									
Maternal schooling									
None/< 8 years	0.91	< 0.0001	1.58 (1.00;2.47	0.47	< 0.0001	2.30 (1.53;3.46)	0.42	0.0278	2.24 (0.62;8.02)
8–11 years	0.33	0.0378	0.88 (0.57;1.36)	0.24	< 0.0001	1.59 (1.07;2.35)	0.23	0.3293	1.39 (0.44;4.37)
≥ 12 years	0.22		1	0.10		1	0.14		1
Previous living children									
1	0.43	0.0340	1.35 (0.96;1.90)	0.24	0.3070		0.25	0.2870	
2	0.63	< 0.0001	1.63 (1.09;2.45)	0.31	0.0060	1.32 (0.96;1.81)	0.45	0.0240	2.69 (1.07;6.79)
≥ 3	1.30	< 0.0001	2.64 (1.82;3.81)	0.52	< 0.0001	1.65 (1.21;2.24)	0.34	0.1040	1.16 (0.41;3.31)
Nenhum	0.31		1	0.21		1	0.15		
Previous deceased children									
≥ 1	0.59	0.2986		0.33	0.1362	1.16 (0.84;1.59)	0.22	0.7627	
None	0.49			0.26		1	0.26		
Health care determinants									
Prenatal visits									
None	1.81	< 0.0001	2.90 (2.01;4.19)	1.17	< 0.0001	3.79 (2.70;5.33)	1.82	< 0.0001	6.97 (2.24;21.70)
1–3	1.03	< 0.0001	1.76 (1.26;2.45)	0.52	< 0.0001	1.81 (1.34;2.45)	0.54	0.0404	1.98 (0.61;6.44)
4–6	0.56	< 0.0001	1.19 (0.88;1.59)	0.35	< 0.0001	1.36 (1.07;1.73)	0.22	0.7916	
≥ 7	0.30		1	0.19		1	0.20		1
Type of delivery									
Cesarean	0.38	0.0004	0.96 (0.74;1.25)	0.24	0.0305	1.39 (1.13;1.70)	0.30	0.2895	
Vaginal	0.58		1	0.29		1	0.21		
Type of Hospital									
Public	0.53	< 0.0001	1.30 (0.79;2.15)	0.31	< 0.0001	1.41 (0.93;2.13)	0.33	0.0932	1.16 (0.38;3.51)
Mixed	0.73	< 0.0001	1.69 (1.02;2.79)	0.35	< 0.0001	1.60 (1.05;2.43)	0.21	0.4613	
Private	0.17		1	0.11		1	0.13		1

*Adjusted model to variables of the same stratum with *P* < 0.20 in the bivariate analysis. *IR*, incidence ratio; *CI 95%*, confidence interval of 95% no population

Regarding gestational age, newborns younger than 22 weeks with extreme low birth weight (500–999 g) had a 92.5% death rate, which decreased to 63.2% with an increase in gestational age from 22 to 27 weeks. Among newborns in this same weight stratum, when presenting a gestation age of 28–31 weeks, there was a greater drop in the percentage of death, indicating a 35.6% mortality. Very low birth weight infants (1000–1499 g) presented a variation in gestational age between 22 weeks and, as a maximum, 36 weeks, and those born at 22–27 weeks had a risk of 1.9 (95% CI, 1.2–2.8) times greater of death in the first year of life, when compared with the reference category (32–36 weeks).

The male gender showed an association with infant mortality in newborns of extremely low birth weight and adequate birth weight strata. Among the biological determinants, the offspring of mothers with color/race in the black/brown/Asian/indigenous category, after the adjusted analysis, presented a higher risk of infant mortality when compared with white in the strata of newborns with insufficient birth weight and ABW. Infants with an Apgar score lower than 7 in the fifth minute of life had a significant association with death before the first year of life in all birth weight strata. A score less than 7 in the fifth minute of life increased proportionally with the birth weight increase of newborns.

Regarding the social determinants, children of mothers with a maternal education of less than 8 years of schooling (reference category, 8–12 years of schooling) had a higher percentage of infant death in the first year of life in all strata of newborns. This low maternal schooling presented a risk of 2.3 (95% CI, 1.5–3.4) for infant mortality even among newborns with adequate birth weight (3000–3999 g). Regarding the number of living children, children of mothers with two, three, or more previous living children, when compared with the reference category, did not present a risk for infant mortality when the newborns were found in the lower extremely low birth weight strata. The number of previous deceased children was not related to infant death in the first year of life in the offspring of mothers of all birth weight strata studied.

Among the determinants of care, the lack of prenatal care is a risk factor for infant mortality in newborns weighing 1500 g or more. The performance of one to three prenatal consultations, when compared with mothers who completed seven or more visits (reference category), demonstrated a risk for infant mortality in the very low birth weight, low birth weight, insufficient birth weight, and adequate birth weight strata. These results reveal a risk of death in the first year of life for most of the birth weight strata when the number of prenatal consultations is below that established by the Brazilian Ministry of Health and WHO, i.e., seven or more visits. Regarding the type of delivery, cesarean delivery, among infants with extremely low birth weight, was a protective factor of 0.7 (95% CI, 0.6–0.8) for infant mortality when compared with vaginal delivery, and 0.7 (95% CI, 0.5–0.9) among newborns in the VLBW stratum. In contrast, among newborns of ABW, cesarean delivery showed a risk of 1.3 (95% CI, 1.1–1.7) times for infant death in the first year of life in relation to vaginal delivery. Birth in a public hospital showed a risk of 1.8 for infant mortality (95% CI, 1.4–2.4) only among newborns of the LBW stratum. Throughout the study period, newborns in private hospitals had a 26.7% occurrence of deaths in the first year of life in the ELBW stratum. This scenario rises to 47.7% in hospitals considered to be mixed and to 65.9% in public hospitals.

## Discussion

The study focused on investigating the relation of birth weight in its various strata, especially in the cases of extreme low weight, very low weight, and low weight, with infant mortality, subject to the influence of the main biological, social, and care-related determinants of health within a time temporal series in a capital in the extreme south of Brazil.

Low birth weight and prematurity are universally recognized as the most important risk factors for neonatal mortality. This research confirmed the importance of specialized assistance to newborns with LBW, as the percentage of deaths in the first year of life among them was high: 55% for children under 1000 g (extremely low birth weight) and 11 % among those from 1000 to 1499 g (very low birth weight). These data are similar to other studies in Brazil, which have shown that infant mortality in these groups is still very high, especially among newborn infants less than 750 g [4, 9, 10].

Different associations between the determinants investigated with infant mortality among newborns of the various birth weight strata during the study period were observed. Those with adequate birth weight (3000–3999 g) had only 0.30% death in the first year of life. Regarding biological determinants, in this same stratum, a lower maternal age (17 years or less) revealed risk, whereas an older age (35 years or more) indicated protection for infant mortality. It should be noted that, among newborns in the ABW stratum, there was an inversely proportional relationship between maternal age and infant death in the first year of life.

The 5-min Apgar Index was shown to be a risk factor for infant mortality in newborns in all birth weight strata, confirming that it is an important indicator of the viability of the newborn during perinatal care. In this context, it should be noted that, among infants in the extremely low birth weight and very low birth weight strata, as well as in the others, an Apgar Index of the fifth minute of life of less than seven meant that birth weight is directly proportional to the risk of infant mortality. Thus, the higher the birth weight, paradoxically, the greater the likelihood of death. As a possible hypothesis of this result, in the conditions of birth weight considered adequate, newborns with an Apgar score of less than seven in the fifth minute of life will have a greater influence of the other determinants of health. In this perspective, a study conducted in the same state in Brazil in 2012, also based on Live Birth Information System, showed that there was a relationship between the prenatal coverage of the pregnant woman and the vitality of the newborn (Apgar Index in the fifth minute of life), as a low number of consultations by the pregnant woman reflected in a low Apgar score for the newborn [11].

In addition to the greater risk for mortality, newborns weighing less than 1000 g, physiologically, present a high risk of visual loss and blindness due to retinopathy of prematurity, pulmonary dysplasia (35–45% of cases), and heart disease [12]. There is also a higher incidence of growth failure, developmental delay, and number of hospital admissions, especially in the first year of life (four times greater likelihood) due to respiratory infections caused by respiratory syncytial virus.

The risk factors for neonatal survival and neonatal mortality in a cohort of live births with birth weight up to 1500 g (corresponding to the low birth weight and very low birth weight scores in the present study) were investigated in the city of São Paulo, the most populous city in Brazil [13]. An Apgar Index of less than seven in the fifth minute of life demonstrated a low vitality of the newborns and was related to a high incidence for infant mortality, concluding that the extremely low birth weight represents an epidemiological parameter of survival for the newborn. Corroborating with these findings, another study, performed at the neonatal intensive care unit in a tertiary hospital in the city of Porto Alegre, analyzed the clinical profile of newborns and observed that all newborns in the ELBW stratum required pulmonary ventilation (72.7% non-invasive and 16.6% high frequency) [14].

Maternal education, number of prenatal consultations, and type of hospital, considered as social determinants, have been shown to influence the relationship between birth weight and infant mortality. This result indirectly reflects the socioeconomic conditions that are related to the quality of perinatal care. In this perspective, some studies pointed out that women with higher levels of education tend to be more prone to taking care of themselves, since, given their greater access to information, they are more aware of the care that must be taken both in the gestational and postpartum periods [11, 15]. According to these authors, this condition is, in general, linked to a unique socioeconomic situation, which contributes to better discernment in health-related decision-making. This could be confirmed, particularly, in Brazil where improvements in education and health care have reduced the risk for low birth weight in the last years [16].

Regarding the determinants of care, prenatal care was an extremely important factor, since, as the number of consultations increased, there was a decrease in the incidence rate for infant mortality, except for newborns in the strata of extremely low birth weight. The importance of prenatal care has already been demonstrated in several studies. The adequate follow-up during pregnancy can assist in the early identification of problems in the fetus, making it possible to act in time to reduce the impact of possible complications, as well as to prevent the births of very low birth weight infants (less than 1500 g) [13] and has a privileged status in the reduction of prenatal and perinatal complications and deaths, thereby becoming a form of compensatory policy aimed at minimizing the effect of socioeconomic inequalities [5]. In this context, in a peculiar way, an epidemiological phenomenon was described by researchers as the paradox of low birth weight [17]. They found that better medical care during pregnancy may lead to the detection of certain conditions, such as intrauterine growth restriction through obstetric interventions that fetuses, probably stillborn infants, become viable, thereby contributing to an increase in the LBW rates.

Another aspect in the scope of care analyzed by this study is the type of delivery. Among the newborns in the strata of extremely low birth weight and very low birth weight, cesarean delivery showed a protective effect for infant mortality. Differently, for newborns in the adequate birth weight stratum, cesarean delivery demonstrated a risk for infant mortality. Regarding the type of delivery, it has been demonstrated in other studies in Brazil that cesarean had a protective effect for neonatal mortality among newborns of VLBW [10, 13]. According to some authors, there are indications that children undergoing chronic hypoxic stress, such as fetuses with restricted intrauterine growth, could have a worse outcome after vaginal delivery due to the risk of further injury from perinatal hypoxia due to labor [18–20]. This has also been proven in a recent survey conducted in China, which investigated the relationship between gestational age and type of delivery in newborn infants under 2500 g. The conclusions stated that the rates of stillbirths and neonatal deaths in term pregnancies were significantly lower in cesarean deliveries than in vaginal deliveries and these same findings were also verified for preterm newborns 7and LBW [21].

Regarding the type of hospital, the results showed a risk for infant mortality among newborns in the extremely low birth weight stratum, which revealed an 85% higher likelihood of death in public hospitals than in private ones, and a 46% higher likelihood of this outcome when compared with mixed and private hospitals. Moreover, newborns weighing less than 1500 g born in public hospitals had a higher risk for infant mortality in the period studied. It should be noted that, in particular, a small increase in newborns in this birth weight stratum has previously been observed in the municipality [22]. In this sense, the 2018–2021 Municipal Health Plan of Porto Alegre has recently indicated the need to increase the number of beds of the neonatal intensive care unit (NICU) in order to achieve an adequate index of satisfaction and that the average number of days in the Neonatal NICU is below the levels recommended in the municipality [22].

It is important to highlight a peculiar and interesting situation that occurs in some major cities of Brazil. More recently, as a way to improve neonatal care, some authors have warned about the increase of judicial decisions to obtain vacancies in neonatal intensive care unit [23]. According to them, because it is a highly complex service that demands large investments and resources, when public needs are not met under the justification of budget constraints, problems have often been aggravated by the progressive deficit in hospitals of the public health network.

In this study, the analysis of a health indicator such as infant mortality (outcome), considering the birth weight distributed among the different strata, allowed an interesting evaluation of the different determinants of health related to infant death. The studies usually aimed only at low birth weight infants (less than 2500 g), not discriminated according to their subcategories (extremely low weight and very low weight), which may induce misinterpretations regarding their association with infant mortality. Therefore, an evaluation of this detailed relationship in the different low birth weight strata, coupled with an analysis of the determining factors, also considered as mediators, should be interpreted as a strong point of this study. In addition, the use of two official health information systems ratified by the Ministry of Health of Brazil (Live Birth Information System and Mortality Information System), available across the national territory and with a high level of quality [24–27] with the specific data of Porto Alegre, may also be considered strengths of this study (Additional file 1).

Some of the limitations of the study that may be pointed out include the low number of newborns in some strata of weight, especially among those with over 4000 g; the use of categorical maternal variables such as gestational age, which, from 2012 onwards, became continuous, and the degree of maternal schooling classified in the Live Birth Information System, at intervals of years of study; and the lack of information about maternal smoking and other gestational diseases and obstetric indication for cesarean delivery that could contribute to the understanding of causal relationships on low birth weight.

Therefore, fundamentally, prenatal care programs should develop effective actions to promote health and strengthen the importance of care during pregnancy. The development of effective strategies promotes fetal growth and development, thus contributing to an adequate birth weight of newborns, and should prioritize the follow-up and management of risk pregnancies and early detection of maternal-fetal diseases.

## Conclusions

The infant mortality rate has shown a declining trend in recent years, but low birth weight infants are still at increased risk for mortality.

Among the determinants of health, lower age and maternal schooling are associated with a higher percentage of infant deaths for all birth weight strata. Insufficient prenatal (less than three visits) demonstrated a higher risk for infant mortality among low birth weight, insufficient, and adequate birth weight infants. The delivery performed in a public hospital presented a higher risk for mortality for the extremely low birth weight infants. Cesarean section was a protective factor for mortality among extremely low and very low birth weight infants and, on the contrary, was a risk factor for newborns with adequate birth weight.

Some of the determinants of health investigated in this study have shown an influence on the birth weight, which is therefore closely related to infant mortality. The determinants of care have proved to be more representative and are liable to a more direct and punctual intervention by the public authorities. Consequently, it is necessary to ensure a more targeted and specific look at newborns weighing less than 2500 g in order to expand and qualify the care focus in prenatal, perinatal, and neonatal care, as well as the preparation and application of efficient policies in the field of maternal and infant health. In this way, it may be possible to reduce the rates of low birth weight, with a subsequent increase in the survival of these infants.

Finally, although low birth weight continues to be an important risk factor for infant mortality, it is necessary to evaluate, individually, the different strata of LBW under the view of the different determinants of health involved. In this way, more effective and specific measures can be performed in the health care of the different LBW categories.

## Supplementary information

**Additional file 1:** Database originated from the link between the two health information systems used (Live Birth Information System and Mortality Information System). It contains all the variables used in the sixteen year period evaluated. (SAV 83644 kb)

## Data Availability

All the data generated by the linkage of the two health information systems and used for the publication of this manuscript are available in the file “Database (Vilanova_CS).sav” attached in the supplementary information files.

## Abbreviations

ABW: Adequate birth weight
AAPC: Average Annual Percentage Change
CI: Confidence interval
ELBW: Extremely low birth weight
HCPA: Hospital de Clínicas de Porto Alegre
IBGE: Brazilian Institute of Geography and Statistics
IM: Infant mortality
IR: Incidence ratio
IW: Insufficient weight
LBS: Live Birth Statement
LBW: Low birth weight
MACR: Macrosomic
NCDs: Non-communicable diseases
NICU: Neonatal intensive care unit
NM: Neonatal mortality
PNM: Postnatal mortality
SIM: Mortality Information System
SINASC: Live Birth Information System
SPSS: Statistical Package for the Social Sciences
UFRGS: Universidade Federal do Rio Grande do Sul
VLBW: Very low birth weight
WHO: World Health Organization

## Footnotes

1 The reference categories were chosen based on the category of lower mortality rate, with the exception of vaginal birth (global consensus) and maternal age of 18–34 years (fertile age).
